# *Terminalia arjuna* in Chronic Stable Angina: Systematic Review and Meta-Analysis

**DOI:** 10.1155/2014/281483

**Published:** 2014-01-30

**Authors:** Navjot Kaur, Nusrat Shafiq, Harish Negi, Avaneesh Pandey, Srinivas Reddy, Harpreet Kaur, Neelima Chadha, Samir Malhotra

**Affiliations:** ^1^Department of Pharmacology, Postgraduate Institute of Medical Education & Research, Chandigarh 160012, India; ^2^Department of Cardiology, Postgraduate Institute of Medical Education & Research, Chandigarh 160012, India; ^3^ICMR Advanced Centre for Evidence-Based Child Health, Advanced Paediatrics Centre, Postgraduate Institute of Medical Education & Research, Chandigarh 160012, India; ^4^Dr. Tulsidas Library, Postgraduate Institute of Medical Education & Research, Chandigarh 160012, India

## Abstract

*Background*. *Terminalia arjuna* is a popular Indian medicinal plant with its bark been used for over centuries as cardiotonic. The bark has been found to contain several bioactive compounds including saponins and flavonoids. A number of experimental and clinical studies have been conducted to explore therapeutic potential of *Terminalia arjuna* in cardiovascular ailments specially in patients of coronary heart disease. A number of narrative reviews have been done but no systematic review has been conducted to date. *Objective*. To systematically review and conduct a meta-analysis on the available literature evaluating the efficacy of *Terminalia arjuna* in patients of chronic stable angina. *Study selection*. We included randomised, pseudo-randomized and before-after comparative studies which compared *Terminalia arjuna*/commercial preparation of *Terminalia arjuna* with current standard/ conventional treatment regimens in patients with chronic stable angina. *Findings*. Studies were found to be of poor methodological design. We found no significant difference in the *Terminalia arjuna* group as compared to control arm in the outcomes for which we were able to pool data and undertake meta-analysis. *Conclusions*. Currently, the evidence is insufficient to draw any definite conclusions in favour of or against *Terminalia arjuna* in patients of chronic stable angina. Further, well-controlled multicentric clinical trials need to be conducted in large number of patients to explore the therapeutic potential of *Terminalia arjuna* if any.

## 1. Introduction

Complementary and alternative medicine (CAM) is very frequently used by patients in various clinical settings (e.g., oncology and hypertension) as has been observed by authors [1, 2]. Very few of these are, however, supported by clinical trials. An attempt has been made by investigators in India to evaluate the efficacy and safety of a commonly used medicinal plant, *Terminalia arjuna*, commonly called Indradru, Arjuna, Dhavala, Nadisarja, Kakubha, Veeravriksha and Partha [3]. It is a large evergreen tree that grows abundantly throughout India and belongs to Combretaceae family.

*Terminalia arjuna* has been known to possess cardiovascular benefits as early as 500 BC and its bark has been used in Indian traditional system of medicine for over three centuries as a cardiotonic [4, 5]. Active constituents of *Terminalia* bark include tannins, cardenolide, triterpenoid saponins (arjunic acid, arjunolic acid, arjungenin, and arjung lycosides), flavonoids (arjunone, arjunolone, and luteolin), gallic acid, ellagic acid, oligomericproanthocyanidins (OPCs), phytosterols, and various minerals such as calcium, magnesium, zinc, and copper [3, 5–7].

*Terminalia arjuna* has been clinically tested in a number of cardiovascular conditions including ischaemic heart disease, [8] hypertension, [9] mitral regurgitation, [10] endothelial dysfunction, [11] and heart failure, [12]. A number of reviews have been done describing the effect of *Terminalia arjuna* in cardiovascular diseases but ordinary reviews have limitations like assessment of publication bias, insufficient information regarding the pooled outcomes, and heterogeneity.

Nine clinical studies have been conducted to study the effects of *Terminalia arjuna* in patients with angina alone [13–21]. No systematic review has been conducted for *Terminalia arjuna* in patients of chronic stable angina. Therefore, we planned to systematically review and conduct a meta-analysis on the available literature evaluating the efficacy of *Terminalia arjuna* in patients of chronic stable angina.

## 2. Methods

### 2.1. Literature Search

Literature search was conducted independently by two authors (Navjot Kaur and Avaneesh Pandey) using electronic databases including PubMed, Embase, and Cochrane and national databases like Council for Research in Ayurvedic Sciences, India, up to May 2013. The search strategy was developed by two authors (Harpreet Kaur and Neelima Chadha) with expertise in library search. We entered the medical subjects heading (MeSH) terms and text words, including “*Terminalia arjuna*,” “heart failure,” “angina pectoris,” “cardiovascular effects,” and “clinical trials” separately and in combination. Manual search was made by going through the reference lists of the retrieved articles to identify additional relevant studies. The search results were pooled and discrepancies were resolved by consensus.

### 2.2. Inclusion Criteria

We included randomised, pseudo-randomized, and before-after comparative studies which compared *Terminalia arjuna*/commercial preparation of *Terminalia arjuna* with current standard/conventional treatment regimens in patients with chronic stable angina. Conventional treatment regimens are comprised of the following drugs: antiplatelet agents, beta blockers, calcium antagonists, nitroglycerine, and other nitrates.

### 2.3. Study Outcomes

- Primary endpoint:
   death or acute myocardial infarction.
- Secondary endpoints:
  - change in the clinical condition (resolution of anginal symptoms, frequency of anginal attacks, and requirement of rescue medication);
  - change in parameters of stress testing:
    - duration of exercise;
    - maximal ST segment depression;
    - time to recovery from ST segment depression and;
    - double product;
  - changes in left ventricular ejection fraction and left ventricular mass;
  - adverse events.

### 2.4. Data Extraction

Data extraction forms were used to obtain the following information: characteristics of study participants, number of participants, type of intervention (dose and duration), randomization, blinding, study outcomes, and duration of follow-up. The data were extracted independently by two investigators (Harish Negi and Avaneesh Pandey) and compiled by a third investigator (Navjot Kaur). Differences were removed by consensus. Authors were contacted if it was felt that data additional to the one published may be useful.

### 2.5. Data Analysis

Continuous data were expressed as mean ± S.D and dichotomous data as *n* (%). We calculated standard mean difference (SMD) between the experimental and treatment arm with 95% CI. Separate forest plots were constructed for pooled study outcomes. Pooling of data was planned if two or more studies had used the same outcome and expressed the data in the format to enable pooling. It was planned to use Mantel-Haenszel model for pooling the data in case of significant heterogeneity and fixed effect model in case heterogeneity is insignificant. It was planned to test for heterogeneity by *I* ^2^ statistics and publication bias by inverted funnel plot.

Quality assessment of included studies was done by the method described in the Cochrane Handbook of Systematic Reviews. Following parameters were considered for quality assessment: presence or absence of factors like randomization, allocation concealment, blinding of participants and study physician, blinded outcome assessment, completeness of outcome data and selective reporting bias. Based on these parameters, the studies were reported as having high, low, or unclear risk of bias.

## 3. Results

**Figure 1:**
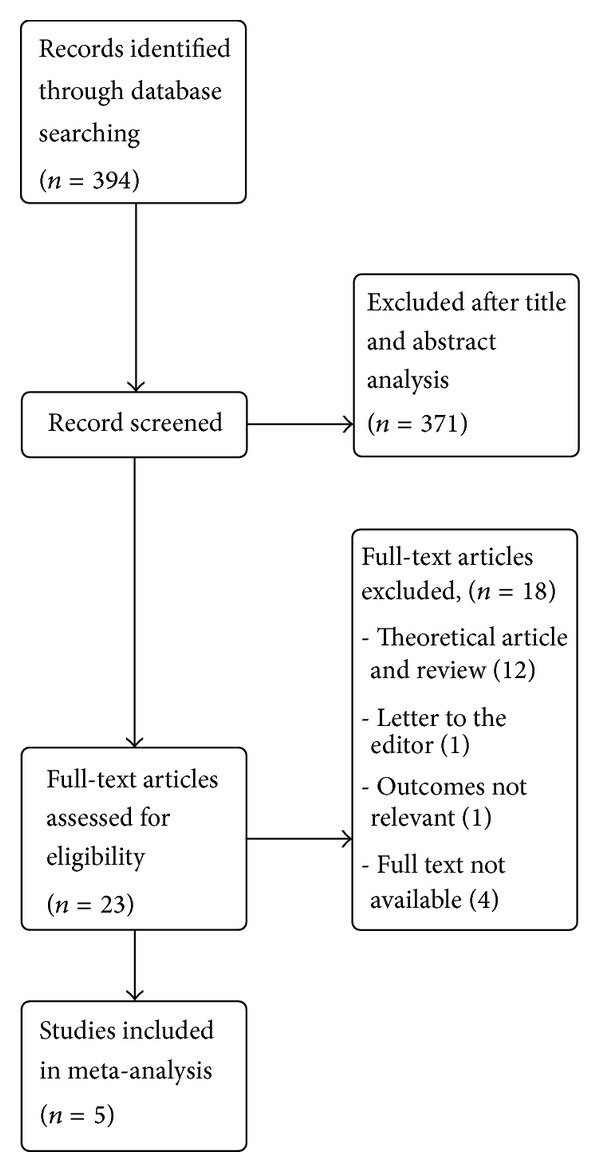
Flow chart of methods.

394 hits were obtained after combining search of all selected databases. After excluding duplicate articles, thorough screening of titles and abstracts, and searching of cross-references, 24 studies were found suitable for full-text search. Out of these, 5 studies were considered for data extraction and quality assessment. Other studies were excluded for the following reasons: 12 studies were review articles, one was letter to editor [10], one was authors reply to comments on the article [22], and one study outcomes were different [23]. Full text was not available for four studies [18–21]. Authors or institute library were contacted by mail but to the time of analysis these were not received by us (Figure 1).

### 3.1. Study Characteristics and Quality Assessment

The included studies were of randomised, pseudo-randomised, and before-after comparative design, with small patient population (ranging from 10 to 58) and had duration of follow-up ranging from 4 weeks to maximum of 12 weeks (see Table 1).

*Terminalia arjuna* was given as tablet, capsule, and in powdered form. Control group were given conventional treatment comprising of antiplatelet agents, beta blockers, calcium antagonists, nitroglycerine, and other nitrates. All the included studies were identified as having high risk of bias as shown (Table 2).

### 3.2. Study Outcomes

*Primary Outcome*. No study reported the outcomes of deaths or acute myocardial infraction.

Secondary outcomes are as follows.

#### 3.2.1. Change in the Clinical Condition

For the endpoint of improvement in clinical condition of the patients (which included resolution of anginal symptoms, frequency of anginal attacks, and requirement of rescue medication), we were not able to pool the data as different studies reported these outcomes in different measures. Bharani et al. in their study reported that therapy with *Terminalia arjuna* leads to improvement in NYHA functional capacity by one class in 27 out of 58 patients (46.55%) as compared to none in the placebo group. They also reported a significant decrease in frequency of angina with reduced need for rescue medication in the *Terminalia arjuna* group as compared to placebo (5.69 ± 6.91 mg/week versus 18.22 ± 9.29 mg/week) whereas no significant differences were observed in these parameters when *Terminalia arjuna* and isosorbide-mononitrate (ISMN) groups were compared [14]. The study by Dwivedi et al. reported amelioration in anginal symptoms with 50% decrease in anginal frequency and decrease in requirement of rescue medication from 4 ± 2 tablets per day baseline to 1.8 ± 1 tablets per day by the end of 3 months of therapy with *Terminalia arjuna* [16]. Subsequent study by the authors showed that patients who received *Terminalia arjuna* had significant reduction in the frequency of anginal attacks per day from 3.50 ± 1.98 at baseline to 1.08 ± 1.08 as compared to 3.10 ± 0.72 at baseline to 1.17 ± 0.84 in patients on conventional therapy alone by the end of 3 months [15]. One study, which compared safety and efficacy of Hartone (a proprietary herbal product mainly containing *Terminalia arjuna*) with isosorbide mononitrate, showed symptomatic relief in 80% of patients on Hartone as compared to 70% in ISMN group [13]. There was reduction in occurrence of anginal attacks from 79/week to 24/week in Hartone group as compared to 26/week to 7/week in ISMN group. In mother clinical trial [17], authors mentioned a marginal reduction in the consumption of sublingual nitroglycerine tablets in patients on Terminalia therapy; however, the data for the same was not provided.

#### 3.2.2. Changes in Parameters of Stress Testing

**Figure 2:**
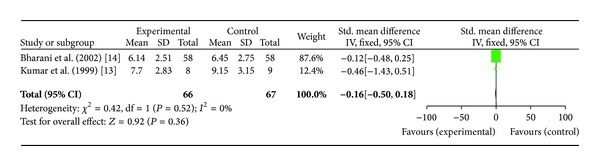
Plot of mean difference of duration of exercise in two studies comparing *Terminalia arjuna* and conventional therapy in patients with stable angina.

*(i) Duration of Exercise.* Two studies [13, 14] including total of 66 patients were included in this analysis. Bharani et al. reported increase in duration of exercise in patients who were started on *Terminalia arjuna* therapy (6.14 ± 2.5 mins versus 4.74 ± 2.34 mins at baseline) as compared to patients in placebo group (4.76 ± 2.38 mins versus 4.74 ± 2.34 mins at baseline) [*P* < 0.005], while conventional therapy was found to be equally effective (6.45 ± 2.75 versus 4.74 ± 2.34 min at baseline). Another study reported [14] increase in duration of exercise from 7.51 ± 2.17 mins at baseline to 7.70 ± 2.83 mins [*P* = *NS*] after 3 months of therapy with Hartone whereas patients in the ISMN group showed a decrease in exercise duration from 6.83 ± 3.17 at baseline to 5.92 ± 2.45 mins by the end of study. However, after pooling data, standard mean difference between the experimental and treatment arm was (−0.16[−0.50, 0.18]; *P* = 0.36), which was not statistically significant as shown in Figure 2.

**Figure 3:**
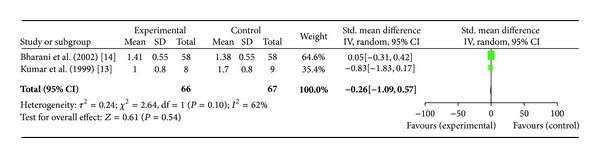
Plot of mean difference of maximum ST segment depression in two studies comparing *Terminalia arjuna* and conventional therapy in patients with stable angina.

*(ii) Maximum ST Segment Depression*. Two studies [13, 14] including total of 66 patients were included in this analysis. There was no significant difference in the SMD in the outcome of maximum ST segment depression (−0.26[−1.09, 0.57]; *P* = 0.54) (Figure 3). Bharani et al. showed significant decrease in maximal ST segment depression (1.41 ± 0.55 mm versus 2.21 ± 0.56 mm at baseline) in *Terminalia arjuna* group versus no change in the placebo group (2.21 ± 0.56 mm as compared to baseline) [*P* < 0.005]. Kumar et al. showed decrease in ST segment depression from 1.2 ± 0.8 mm at baseline to 1.0 ± 0.8 mm in patients after Hartone therapy whereas in ISMN group there was increase in ST segment depression from 1.5 ± 1.0 mm at baseline to 1.7 ± 0.8 mm by the end of study.

**Figure 4:**
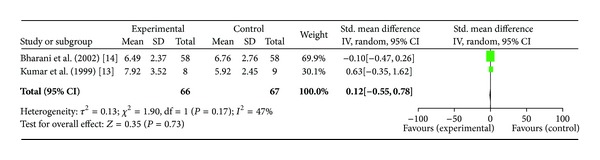
Plot of mean difference of time to recovery from ST segment depression in two studies comparing *Terminalia arjuna* and conventional therapy in patients with stable angina.

*(iii) Time to Recovery from ST Segment Depression*. Two studies [13, 14] including total of 66 patients were included in this analysis. For the outcome of time to recovery from ST segment depression in minutes, no significant difference was found between experimental and the control arm with SMD (0.12[−0.55, 0.78]; *P* = 0.73) (Figure 4).

Bharani et al. showed decrease in recovery time in *Terminalia arjuna* group (6.49 ± 2.37 mins versus 9.01 ± 3.40 at baseline) as compared to placebo group which showed increase in this parameter (9.27 ± 3.39 min versus 9.01 ± 3.40 at baseline) while recovery time for conventional therapy was 6.76 ± 2.76 min. Another study reported increase in recovery time in Hartone group from 7.72 ± 1.36 mins at baseline to 7.92 ± 3.52 mins at the end of 3 months whereas the corresponding change in ISMN group was decrease from 6.84 ± 3.17 mins at baseline to 5.92 ± 2.45 mins by the end of study.

**Figure 5:**
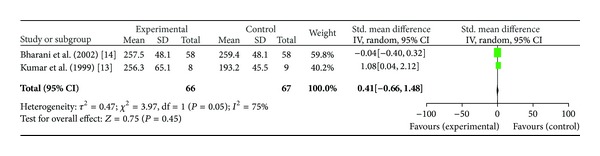
Plot of mean difference of double product in two studies comparing *Terminalia arjuna* and conventional therapy in patients with stable angina.

*(iv) Double Product*. Two studies, [13, 14] including total of 66 patients were included in this analysis. No significant difference was found in the outcome of double product; SMD (0.41[−0.66, 1.48]; *P* = 0.45 as well. (see Figure 5). Bharani et al. reported higher double product after *Terminalia arjuna* therapy (257.5 ± 48.1 versus 224.6 ± 4.48 at baseline) as compared to placebo group (231.1 ± 48.3 versus 224.6 ± 44.8 at baseline) and similar effect with conventional therapy (259.4 ± 4.81 versus 224.6 ± 4.48 at baseline). Another study showed higher double product in Hartone group (256.3 ± 65.1 versus 234.7 ± 37.8 at baseline) as compared to ISMN group which showed a decrease in double product (193.2 ± 45.5 versus 194.1 ± 58.0 at baseline).

#### 3.2.3. Left Ventricular Ejection Fraction and Left Ventricular Mass

One study reported that therapy with *Terminalia arjuna* over a period of 3 months led to significant increase in left ventricular ejection fraction (LVEF) (from 42.25 ± 9.96% at baseline to 52.57 ± 12.32%; *P* < 0.01) and decrease in left ventricular mass (from 159.18 ± 51.11 g/m^2^ at baseline to 127.47 ± 52.40 g/m^2^; *P* < 0.01) at the end of study. The changes in the control group for LVEF and LVM at the beginning and end of follow-up for 3 months were 51.83 ± 5.99% at baseline to 49.83 ± 2.52% and 159.11 ± 38.92 g/m^2^ at baseline to 160.78 ± 54.23 g/m^2^, respectively [15]. In another study left ventricular ejection fraction measured as LVEF% was found to be significantly enhanced as compared to pretreatment values (from 42.33 ± 6.97% to 46.16 ± 4.66%) in patients of stable angina [17].

### 3.3. Adverse Events

A total of 15 adverse events were reported in 2 studies including a patient population of 78. Adverse events included constipation in 4 patients, headache in 3, body ache in 3, abdominal discomfort in 2, dizziness, pedal edema, and transient cardiac failure each in one patient.

### 3.4. Publication Bias

The funnel plot could not be constructed as there was insufficient information for its construction that could be obtained from these studies.

## 4. Discussion

This systematic review examined and compared the effectiveness of *Terminalia arjuna* in patients with chronic stable angina alone or in combination with conventional therapies. Unfortunately, none of the included studies reported data on the selected primary outcome of death or acute myocardial infarction. Though the studied outcomes such as frequency of angina symptoms, NYHA functional class, and ECG abnormalities do provide prognostic information if these parameters were integrated into some scoring systems, for example, Duke treadmill score [24] probably that would have provided more clinically useful information. We found no significant difference in the *Terminalia arjuna* group as compared to control arm in the parameters of exercise duration, time to maximal ST segment depression, and time to recovery and double product.

The study designs were found to be compromised perhaps due to inadequate resources. There was lack of proper randomization procedure, lack of allocation concealment, and blinding procedures. Moreover, sample size was uniformly inadequate across studies with maximum sample size of only 58 patients. As per the epidemiological data current event rate of adverse cardiovascular events (including CV death, MI, or stroke) in patients of chronic stable heart disease is about 4% [24]. In order to decrease this event rate to 3% through an intervention, it would need to be tested in two-arm clinical trial with 7207 patients in each arm, keeping in consideration an alpha value of 0.05 and 90% power. This would mean that, to evaluate the impact of any intervention on hard cardiovascular endpoints in patients with stable CHD, approximately 14,000 patients would need to be enrolled. For attaining the target sample size, multicentric studies would need to be conducted which would require huge amount of resource inputs.

Also none of the included studies provided sufficient details regarding the source of *Terminalia arjuna*, botanical identification, method of extract preparation, and standardization of the finished product. Similar observation has been made in other study also [25]. Characterisation and standardisation of the medicinal plants along with stability testing is important in case of clinical trials with traditional medicines [26].

Thus it is evident from present study that currently the evidence is insufficient to draw any definite conclusions in favour of or against *Terminalia arjuna* in patients of chronic stable angina. To ensure that a potential therapeutic molecule might not go into disrepute just because of drawing conclusions from underpowered studies and on the other side a molecule of no therapeutic value be promoted on the basis of studies which are not sufficiently powered, well-controlled multicentric clinical trials need to be conducted in large number of patients to explore the therapeutic potential of *Terminalia arjuna* in future.

## Figures and Tables

**Table 1:** Characteristics of included studies.

S. no.	Author	Sample size	Population studied	Study design	Duration of follow-up	Test group	Control group
(1)	Bharani et al., 2002 [14]	58	Male with stable angina (NYHA class II-III)	Randomized crossover placebo controlled trial	4 weeks	*Terminalia arjuna* (500 mg TDS)	Isosorbide mononitrate (20 mg BD)

(2)	Kumar et al., 1999 [13]	20	Adult with stable angina	Open comparative trial	12 weeks	Hartone 2 cap BD for 6 months, 1 cap BD for next 6 months	Isosorbide mononitrate (20 mg BD)

(3)	Dwivedi and Jauhari, 1997 [15]	24	Adult with stable angina (NYHA-IV)	Randomised controlled trial	12 weeks	Conventional + *Terminalia arjuna *	Conventional

(4)	Dwivedi and Agarwal, 1994 [16]	20	Adult with stable and unstable angina	Before and after treatment comparison	12 weeks	500 mg *Terminalia arjuna* BD	—

(5)	Jain et al., 1992 [17]	25	Adult with stable angina	Before and after treatment comparison	12 weeks	Conventional + *Terminalia arjuna* 500 mg BD	—

**Table 2:** Quality assessment of included studies.

Study ID	Randomization	Blinding of participants and personnel	Allocation concealment	Blinded outcome assessment	Incomplete outcome data	Selective reporting
Bharani et al., 2002 [14]	**✓**	**✓**	NS	NS	NS	NS
Kumar et al., 1999 [13]	**✓**	NS	NS	NS	NS	NS
Dwivedi and Jauhari, 1997 [15]	**✓**	NS	NS	NS	NS	NS
Dwivedi and Agarwal, 1994 [16]	NS	NS	NS	NS	NS	NS
Jain et al., 1992 [17]	NS	NS	NS	NS	NS	NS

NS: not specified.
